# Three evolutionary radiations shaped the evolution of global religious diversity

**DOI:** 10.1017/ehs.2025.10027

**Published:** 2025-11-17

**Authors:** Anastasia Ejova, Oliver Sheehan, Remco Bouckaert, Simon J. Greenhill, Jan Krátký, Silvie Kotherová, Jakub Cigán, Eva Kundtová Klocová, Radek Kundt, Joseph Watts, Joseph Bulbulia, Quentin D. Atkinson, Russell D. Gray

**Affiliations:** 1 School of Psychology, The University of Adelaide, Adelaide, SA, Australia; 2 Department of Linguistic and Cultural Evolution, Max Planck Institute for Evolutionary Anthropology, Leipzig, Germany; 3 School of Computer Science, Faculty of Science, University of Auckland, Auckland, New Zealand; 4 School of Biological Sciences, Faculty of Science, University of Auckland, Auckland, New Zealand; 5 LEVYNA Laboratory for the Experimental Research of Religion, Masaryk University, Brno, Czech Republic; 6 Department of Sociology, Andragogy and Cultural Anthropology, Faculty of Arts, Palacky University, Olomouc, Czech Republic; 7 School of Psychology, Speech and Hearing, University of Canterbury, Christchurch, New Zealand; 8 School of Psychology, Victoria University of Wellington, Wellington, New Zealand; 9 School of Psychology, Faculty of Science, University of Auckland, Auckland, New Zealand

**Keywords:** religion, denominations, diversification, Reformation, computational phylogenetic modelling

## Abstract

Religious diversity has had profound consequences in human history, but the dynamics of how it evolves remain unclear. One unresolved question is the extent to which religious denominations accumulate gradually or are generated in rapid bursts associated with specific historical events. Anecdotal evidence tends to favour the second view, but quantitative evidence on a global scale is lacking. Phylogenetic methods that treat religious denominations as evolving lineages can help to resolve this question. Here we apply computational phylogenetic methods to a purpose-built data set documenting 291 religious denominations and their genealogical relationships to derive dated phylogenies of three families of world religions – Indo-Iranian, Islamic, and Judeo-Christian. We model the birth of new denominations along the branches of these phylogenies, test for shifts in the birth rate, and draw tentative links between the shifts we find and religious history. We find evidence for birth rate shifts in the Islamic and Judeo-Christian families, corresponding to at least three separate events that have shaped global religious diversity.

## Graphical abstract

**Figure:**
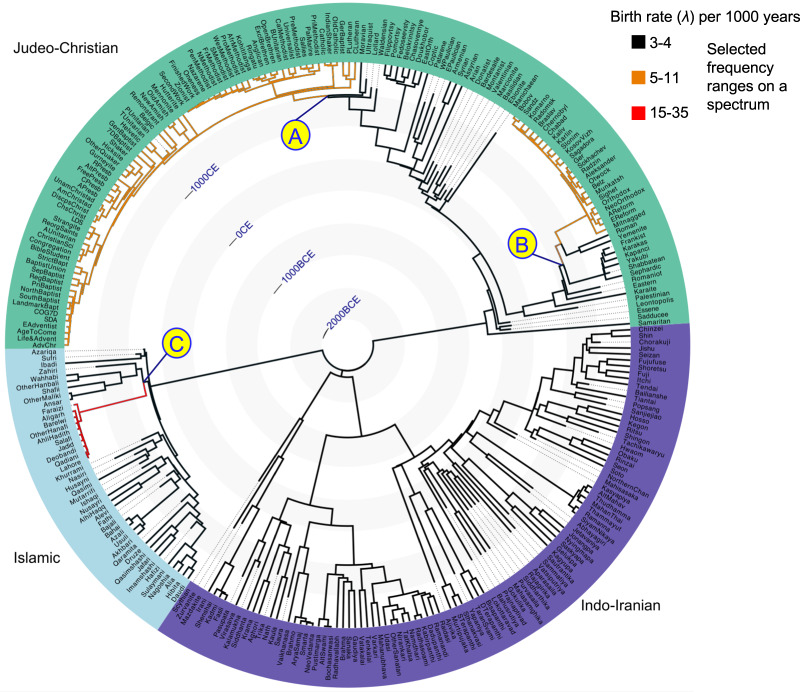

## Social media summary

We uncover rapid bursts in religious denomination growth linked to known history of Islamic and Judeo-Christian families.

Religious diversity, characterised as intergroup differences in religious belief and practice, has had profound consequences throughout human history, fuelling conflict and withstanding attempts to impose homogeneity (Marty, 2011). However, the dynamics of how it evolves remain unclear. One unresolved question, with parallels to the debate between ‘catastrophists’ and ‘uniformitarians’ in the nineteenth century (see Gould, 1987), is the extent to which religious diversity accumulates gradually or in rapid bursts associated with specific historical events. Anecdotal evidence tends to favour a ‘catastrophist’ view. Within Christianity, which claims more adherents than any other religious tradition, a disproportionate amount of religious diversity can be traced to the Protestant Reformation of the sixteenth century, when a large swathe of Christendom rejected papal authority (McGrath & Marks, 2004). Similar events have been described in other religious traditions, a few examples being the *bhakti* movement of medieval and early modern India (Ranade, 1900), the ‘Jewish Enlightenment’ of the eighteenth century (Gottlieb, 2021), and the Kamakura period in Japanese Buddhism (Hara, 1920). However, the quantitative impact of the Reformation and similar events on global religious diversity remains unclear.

Analogies have long been noted between biological evolution and cultural evolution, which includes the evolution of religion (Wilson et al., 2017). As is the case for biological species, new religious denominations usually arise through the fission of existing ones (Finke & Scheitle, 2009), making it possible to model religious diversification using a phylogenetic approach that treats denominations as evolving populations. This approach has already been used to answer a number of questions about the evolution of specific religious beliefs and practices (see e.g. Basava et al., 2021; Matthews, 2012). Episodes of rapid religious diversification also have parallels in evolutionary biology. Evolutionary radiations – ‘the dramatic proliferation of taxa in a clade’ – may have generated most of the diversity that can be observed in plants and animals today (Simões et al., 2016, p. 27). Phylodynamic birth–death models, which reconstruct speciation and extinction rates along phylogenies, allow these radiations to be formally identified (Alfaro et al., 2009; Pennell & Harmon, 2013; Russel et al., 2019). Here we use phylogenetic methods to reconstruct patterns of diversification and test for evolutionary radiations in three major groups of world religions.

‘World religion’ is not a ‘technical’ term, but one whose meaning ‘is largely determined by conventional practice’ (Masuzawa, 2005, p. 9800). Initially applied to religious traditions thought to have ‘universal aspirations’, it later came to apply more loosely to ‘any numerically significant religions’ (Masuzawa, 2005, p. 9803). There is some variation in which religious traditions are assigned to this category, but Buddhism, Christianity, Islam, Judaism, and Hinduism, known as the ‘Big Five’, are almost always included. The so-called ‘world religions paradigm’ has been criticised on various grounds, including arbitrariness, ethnocentrism, and reification, but remains highly influential (Cotter & Robertson, 2016). The field of religious studies has tended to focus on these traditions at the expense of what are known today as ‘indigenous religions’; a tendency that has been criticised (see e.g. Olupona, 2004). However, the demographic dominance of the world religions justifies an emphasis on them when approaching global religious diversity today. As of 2015, adherents of the Big Five alone were estimated to make up 77% of the world’s population, and 92% of those who were religiously affiliated (Hackett & McClendon, 2017).

Using encyclopaedias and other sources, we assembled a purpose-built data set documenting a wide range of religious denominations belonging to the Big Five world religions and their relatives, along with their genealogical relationships, then applied computational phylogenetic modelling to this data to derive dated phylogenies of three religious ‘families’: ‘Indo-Iranian’, ‘Islamic’, and ‘Judeo-Christian’. We modelled rates of religious diversification along the branches of these three phylogenies, tested for shifts in the rate at which new denominations were born, and inferred the likely locations on our phylogenies of the shifts we identified. Finally, we evaluated the fit of our results with known religious history, and made some tentative inferences about the factors that may have driven these shifts.

## Methods

**Selection of units of analysis, religious traditions, and time frame**. Religious groups exist at various scales. The highest-level groupings are typically called ‘religions’ or ‘religious traditions’. Most if not all religious traditions can be divided into a multitude of subgroups for which nomenclature varies, but which in Christianity are typically called ‘churches’, ‘denominations’, or, more pejoratively, ‘sects’. We refer to the taxa that make up our phylogenies as ‘denominations’, but emphasise that this is simply a convenient label for a diverse array of groupings rather than a technical term. It is also worth noting that we treat denominations as social groups distinguished at least partly by religious differences, not as sets of religious doctrines and practices. This is reflected in the names we assign to the denominations in our sample – for example, ‘Anglicans’ rather than ‘Anglicanism’.

In selecting a sample of denominations, we began by focusing on those conventionally classified as belonging to the ‘Big Five’ world religions (Buddhist, Christian, Hindu, Islamic, and Jewish). Ultimately, we expanded our sample to include denominations from 10 additional traditions that are known to be historically related to one or more of the Big Five, but are usually considered ‘religions’ in their own right: Ājīvika, Babī, Bahā’ī, Druze, Jain, Manichaean, Samaritan, Scythian, Sikh, and Zoroastrian. Genealogical relationships between the denominations in our sample allowed us to group them into three religious ‘families’, which we labelled ‘Indo-Iranian’ (Ājīvika, Buddhist, Hindu, Jain, Scythian, Sikh, and Zoroastrian), ‘Judeo-Christian’ (Christian, Jewish, Manichaean and Samaritan), and ‘Islamic’ (Babī, Bahā’ī, Druze, and Islamic *sensu stricto*). We decided at the outset to include only denominations that emerged in 1918 or earlier. This decision was made for two reasons. First, we wanted to maintain a manageable number of taxa in view of the explosive growth of independent churches in Africa and elsewhere during the twentieth century (Barrett et al., 2001). Second, we suspected that religious history over the past 100 years would be difficult to represent using a phylogenetic approach. Phylogenies can represent cladogenesis (the splitting of one lineage into two or more) but not hybridisation (the fusion of two or more lineages into one). Although the religious equivalent of hybridisation has undoubtedly occurred occasionally throughout history, cladogenesis appears to have been far more common (Finke & Scheitle, 2009). However, the ecumenical movement of the twentieth century led to the merging of previously independent Protestant denominations on an unprecedented scale during the twentieth century (Melton, 2005), making a phylogenetic representation untenable.

**Data collection.** We consulted encyclopaedias and other sources and compiled three data sets – one for each religious ‘family’. These data sets consist of lists of denominations with accompanying data. We used general encyclopaedias of religion (Doniger, 2006; Jones, 2005) for all data sets, and more specific encyclopaedias for individual data sets. Additional denominations described in sufficient detail in these more detailed sources were also included in our sample (see our Zenodo repository, https://doi.org/10.5281/zenodo.17092663, ‘Raw data’). Because of our reliance on encyclopaedias and other secondary sources, the denominations that appear in our phylogenies are those delimited by other scholars rather than by ourselves. Our role was limited to the selection of sets of groups that were religiously distinctive and mutually exclusive.

We excluded groups that appeared to be ethnically or politically but not religiously distinctive (e.g. African-American Baptists, Russian Orthodox), as well as those that were clearly subordinate parts of larger entities (e.g. Benedictines). Some of the remaining groups overlapped and intersected in such a way that obtaining a discrete set of taxa required us to prioritise some types of group over others. For example, the Buddhist *Saṅgha* (community of monks) became divided early on into *nikāyas* (‘sects’ or ‘fraternities’) and *vādas* (‘schools’). *Nikāyas* were groups of monks sharing a common set of rules, whereas *vādas* were ‘schools of thought’ whose adherents could belong to any number of *nikāyas* (Harvey, 2013). Including both would have violated the assumption of mutual exclusivity which is fundamental to phylogenetics (De Queiroz & Gauthier, 1992). Because *nikāya* membership was more formalised and definite than adherence to a *vāda*, we ultimately chose to include *nikāyas* and exclude *vādas* (except those that were closely linked to a single *nikāya*). Final lists of denominations for each phylogeny were agreed upon by between two and three coders.

For each denomination that we decided to retain, we gathered information about each that would allow a dated phylogeny to be constructed: parentage, upper and lower bounds of birth date, and, if applicable, upper and lower bounds of extinction date. We defined the parent of a denomination as the denomination to which its founding members were most closely affiliated immediately prior to its birth. Assigning a parent was straightforward in cases where all of the founding members came from the same denomination (e.g. Lutheran, whose founding members were all Catholics). Where the founders were more heterogeneous, we considered the previous denomination of the most influential founding member, or members, to be the parent. For example, the earliest Adventist missionaries came from a range of backgrounds, and the limited data available suggests that a plurality had been Methodists. However, the central figure of the movement, William Miller, had been a Baptist, so Baptists were assigned as the parent denomination in our data set (Knight, 2010). In a handful of cases no single denomination had clear predominance among the founding members (e.g. Rādhāvallabhi, Ḥanbalī, Regular Baptists), but in all such cases the parental populations were closely related enough to treat the relationship as a polytomy (see also *Construction of phylogenies*). The birth date was defined as the point at which the group became distinct enough from its parent (or parents) to qualify as a new denomination. Usually this was an interval (e.g. 800–900 CE), but in some cases it was a single year (e.g. 845 CE), in which case the upper and lower bounds around the birth date were the same. Extinction dates were also collected for denominations that no longer existed in 1918. We distinguished each extinct taxon as either ‘extinct’ in a strict sense or ‘pseudo-extinct’. ‘True extinction’ occurs when an evolutionary lineage terminates, whereas ‘pseudoextinction’ is observed when taxonomists divide a continuous lineage into discrete taxa (Purvis, 2008). The difference can be illustrated by considering the Sadducees and Pharisees, two Jewish ‘sects’ or ‘schools of thought’ (*haireseis*) during the Second Temple period. Both of these names disappeared from the historical record after the Temple’s destruction, but whereas the Sadducees had no known successors, the Pharisees laid the foundations of later Rabbinic Judaism (Efron et al., 2013). It is worth emphasising that beyond choosing denominations that were (or could reasonably be assumed to be) religiously distinctive, we did not systematically collect information on religious doctrines or practices. These characteristics are likely to have been transmitted both vertically (along lineages) or horizontally (between lineages) within our phylogenies, and so the denominations that most closely related in our phylogenies need not always be the most similar in other respects.

**Construction of phylogenies.** We constructed three independent samples of phylogenies corresponding to the three religious ‘families’ documented in our three data sets. For convenience, we labelled these families ‘Indo-Iranian’, ‘Islamic’, and ‘Judeo-Christian’. Each family constitutes a ‘crown group’ – that is, a group containing all known descendants of the last common ancestor of all extant taxa (Jefferies, 1979).

The Judeo-Christian family is the largest, with 143 denominations, 118 of which still existed in 1918. As the label suggests, the vast majority can be distinguished as either Jewish or Christian. However, one (Ebionite) could be characterised as both Christian and Jewish, and two (Manichaean and Samaritan) are usually considered independent religions. All of these religions descend at least in part from the religion of the ancient Israelites, although other influences were also present. For example, the Manichaeans emerged from a Jewish Christian milieu in the third century CE, but Mani and his followers were also influenced by Zoroastrianism and other traditions (Van Oort, 2005).

The Indo-Iranian phylogeny is the second largest, with 103 denominations (71 extant). The name refers to the Indo-Iranian branch of the Indo-European language family, and reflects shared descent from a proto-Indo-Iranian religious tradition that can be partly reconstructed from early Indo-Aryan and Iranian sources (Gnoli, 2005). Other influences are also present, perhaps to a greater extent in this phylogeny than the others. Flood (1996, p. 23) describes Hinduism specifically as having arisen through interaction between Aryan and ‘non-Aryan or Dravidian and tribal cultures, though it is Aryan culture which has provided the “master narrative”, absorbing and controlling other discourses’. The phylogeny is divided into two major branches. One represents Iranian religions: Zoroastrianism and its offshoots, along with the ethnic religion of the Scythians. The other branch consists of the Indo-Aryan religious traditions, sometimes known as the ‘Dharmic religions’ (see e.g. Frawley, 1992). The Indo-Aryan branch is by far the more prolific of the two consisting of Buddhist, Hindu, Jain, and Sikh denominations, as well as the extinct Ājīvikas.

The Islamic phylogeny is the smallest, with 45 denominations (32 extant). Although all the denominations in this phylogeny descended ultimately from the early Islamic community, some offshoots of the Shiite tradition no longer claim to be Muslim: Druze (Madelung, 2005), and Bābī (Azalī and Bahāʾī; Hutter, 2005) are generally considered independent religions. The same may be true of the Imam-shāhī or Satpanthi, though sources on these two groups are less detailed (Madelung, 2005). Like Basava et al. (2021), we exclude both theological schools and Sūfī orders from our phylogenies because of their tendency to cross-cut denominational boundaries.

We considered combining the Judeo-Christian and Islamic trees into a single ‘Abrahamic’ tree. Christians, Muslims, and Jews all claim continuity between their religions and that of Abraham, and although the historicity of Abraham himself has never been established (Van Seters, 2005), the geographic proximity and linguistic relatedness of the ancient Hebrews and pre-Islamic Arabs make a common origin for the Abrahamic religions highly plausible. Proto-Central Semitic, the common ancestor of Hebrew and Arabic, is thought to have diversified between 3,650 and 5,800 years ago in the north-eastern Levant (Kitchen et al., 2009). This language community may have corresponded to a ‘proto-Abrahamic’ religious tradition, but this is speculative. Whereas key features of the ancestral Indo-Iranian religion have been reconstructed from early Indo-Aryan and Iranian sources (Gnoli, 2005), we are not aware of any comparable attempts to reconstruct the ‘Proto-Abrahamic’ or ‘Proto-Central Semitic’ religion. Furthermore, many of the more obvious parallels between the Judeo-Christian and Islamic tradition (e.g. references to Jesus in the Koran) clearly reflect later cultural diffusion rather than common ancestry (Armstrong, 2005).

The process of building the trees began with the selection of a set of ‘tips’ from each data set. The tips of each phylogeny were denominations that either still existed in 1918 or were truly extinct. Denominations that had disappeared through pseudoextinction were not included as tips, because other tips were continuations of them. The next step was to group these tips into clades on the basis of their descent from a common ancestor, and assign an age range to each clade derived either from the birth dates of the denominations within it or from other information from the same sources. Using these data, we compiled data files specifying the denominations within each clade and a set of age-range calibrations on each clade. To allow for overlaps between older and younger clades in some posterior trees, it was convenient to specify normal prior distributions over clade ages in terms of a mean (1918 minus range midpoint) and SD (range divided by 5.152, in line with an assumption that date ranges represented a 99% confidence interval). When the same year was specified as the lower and upper bound of the range, the SD was set to 0.1. Polytomies – cases where multiple denominations were born from the same parent at the same time – were common and were randomly resolved during phylogenetic modelling. Specifically, as is shown in detail in a supplementary figure (https://doi.org/10.5281/zenodo.17092663, ‘Raw data: Constraint trees’), the Judeo-Christian calibration contained 143 tips and 142 internal nodes, 41 (27%) of which were unconstrained. The Indo-Iranian calibration contained 103 tips and 102 internal nodes, 34 of which (33%) were unconstrained. The Islamic calibration contained 45 tips and 44 internal nodes, 18 of which (41%) were unconstrained. We used a Bayesian phylogenetic modelling approach that returned a posterior probability distribution of trees, integrating over the uncertainty in the positioning of these denominations.

**Bayesian phylogenetic modelling.** Although a great deal is known about the genealogies of religious denominations, there can be uncertainty about their exact historical relationships as well as their birth and/or extinction dates. To integrate inferences over this uncertainty, we used a Bayesian phylogenetic method (Bouckaert et al., 2019) to generate a posterior sample of phylogenies constrained by known religious history for each family. Our multi-rate tree prior allowed birth rates to change at arbitrary points along branches of a tree. There were two priors: the rate at which rate changes occur, and the number of possible rates (Barido-Sottani et al., 2020). We tuned the rate change prior so that *a priori* there was a rate change in about half of the trees in each set of phylogenies. We used a Poisson distribution with a mean of 2 for the number of possible rates. This decision was made in order to avoid a situation where there were more rates than rate changes, meaning that some rates might have remained unused in our particular sample of denominations. We assumed constant extinction rates within each tree, and estimated these rates using a log-normal (0, 2) prior, which provided a wide range: a 95% highest probability density (HPD) interval of 0.019–50.4. Birth rates were estimated under a log-normal (−2, 2) prior, which also provided a wide range (95% HPD of 0.0027–6.82). In our main analysis, we assumed that 50% of extinct denominations and 80% of denominations that still existed in 1918 were represented within each phylogeny. Sampling times for the leaf nodes were used as priors. The likelihood consisted of node calibrations encoding timing information and clade constraints based on the historical record. Full details on priors and likelihoods are provided in the supplement (see https://doi.org/10.5281/zenodo.17092663, ‘BEAST’).

Phylogenetic inference and model comparison were performed with BEAST v2.6.3 (Bouckaert et al., 2019) using nested sampling (Russel et al., 2019). We compared the null hypothesis (H0) of no rate changes against the hypothesis of at least one rate change (H1) by restricting the number of rate changes to 0 for H0 and to the range 1–10 for H1. We ran nested sampling with 500 points to obtain a sample from the posterior. Nested sampling works by taking a number of samples from the prior (500 in our case) and iteratively updating the point with the lowest likelihood with a sample from the prior conditioned on having a larger likelihood. We ran four replicates and compared them in Tracer (Rambaut et al., 2018) to ensure that the posterior samples were all from the same distribution, and to gain confidence that the nested sampling analyses had converged. Files containing the posterior samples are available via our Zenodo repository, https://doi.org/10.5281/zenodo.17092663, ‘Posterior samples’.

**Figure 1.**
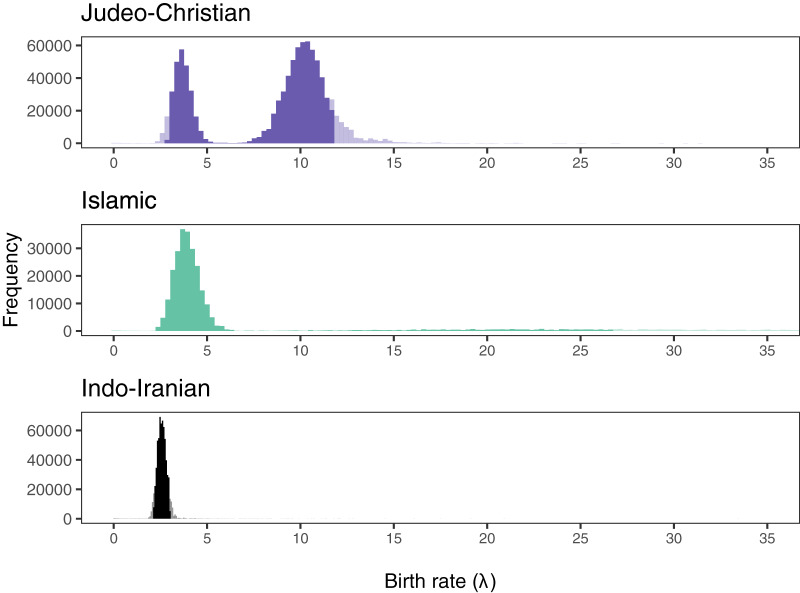
Histograms of birth rate (λ) per 1,000 years from the posterior probability distributions of each religious tradition. The 95% highest posterior density interval is shown in dark colours, while the lightened areas indicate values outside that range.

**Figure 2.**
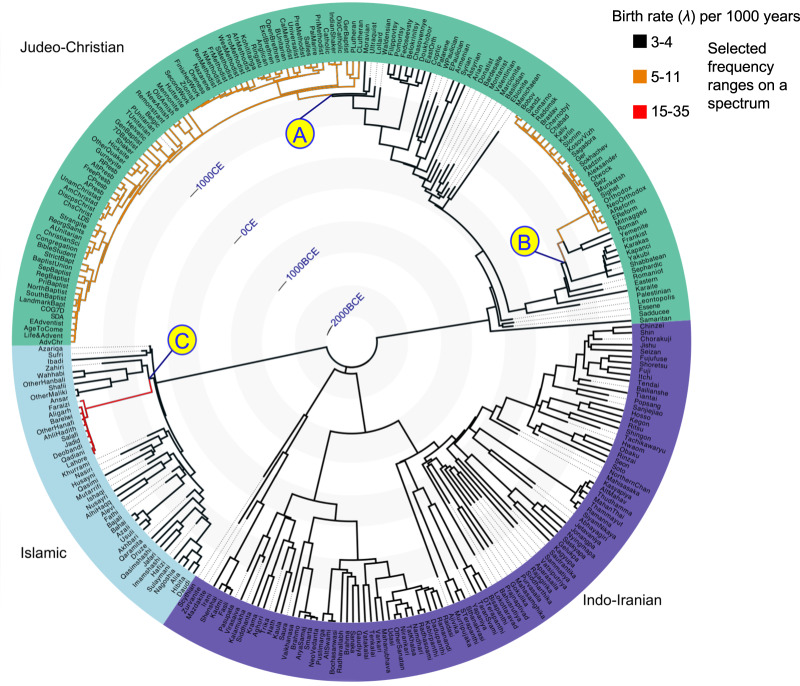
MCC trees for the three posterior samples of phylogenies obtained by nested sampling. The branches are colour-coded by mean birth rate. The letters (A–C) mark the branches along which birth rate shifts were inferred. For finer resolution, see our Zenodo repository, https://doi.org/10.5281/zenodo.17092663, ‘Global tree figure’.

**Visualisation of birth rate frequencies and mean birth rates.** We removed the first 10% of the posterior as burn-in and then plotted the frequencies of birth rates across the posterior at intervals of one lineage split per 1,000 years (see Fig. 1). We used visual inspection to identify clusters of rates that could be assumed to correspond to birth rates before and after a shift. In accordance with the results of our Bayesian phylogenetic analyses, we observed a bimodal distribution of birth rates in the Judeo-Christian and Islamic trees (where there was evidence for a birth rate shift) but not in the Indo-Iranian tree (where there was no evidence for such a shift). We plotted the mean posterior birth rates for each posterior sample of phylogenies (*n* = 3600) on a maximum clade credibility (MCC) tree (see Fig. 2), and used visual inspection to identify the likely locations of birth rate shifts.

**Sensitivity analysis.** To test the robustness of our results to differences in the undocumented number of extinct denominations, we ran four sets of Bayesian phylogenetic analyses with different values for the parameter *sigma*. This parameter, which represents the proportion of extinct denominations included in the sample, was assigned values of 0.99, 0.50, 0.25, and 0.10, whereas *ρ* (the proportion of extant denominations included) was held constant at 0.80. We plotted birth rate frequencies using histograms and mean birth rates on MCC trees as described in the section above, and compared the results. Histograms and MCC trees are presented in our Zenodo repository, https://doi.org/10.5281/zenodo.17092663, ‘Sensitivity analysis’.

## Results

**Phylogenies.** We grouped the retained denominations into three independent families based on their genealogical relationships: ‘Indo-Iranian’ (consisting of Ājīvikas, Buddhists, Hindus, Jains, Scythians, Sikhs, and Zoroastrians), ‘Judeo-Christian’ (Christians, Jews, Manichaeans and Samaritans), and ‘Islamic’ (Babīs, Bahā’īs, Druze, and Muslims *sensu stricto*). The families included a total of 291 denominations (103 Indo-Iranian, 45 Islamic, and 143 Judeo-Christian), of which 220 (71 Indo-Iranian, 32 Islamic, and 118 Judeo-Christian) still existed in 1918. MCC trees based on each of these three posterior samples jointly represent 139,542 years of cultural evolution: 81,583 for Indo-Iranian, 18,746 for Islam, and 39,213 for Judeo-Christianity (see Fig. 2, as well as our Zenodo repository, https://doi.org/10.5281/zenodo.17092663, ‘Maximum clade credibility trees’).

**Bayesian modelling of birth rates and tests for birth rate shifts.** To investigate possible shifts in the diversification rate, we modelled birth and extinction rates for the phylogenies within each posterior sample. We used a multirate tree prior, which allows different birth rates in different parts of each phylogeny. In order to minimise problems with identifiability (see e.g. Morlon et al., 2022), in our main analysis, we assumed that the extinction rate was constant, and that each phylogeny captured approximately 50% of extinct denominations and 80% of denominations that still existed in 1918 (see *Sensitivity analysis* for discussion of the implications of these assumptions).

We found evidence for at least one birth rate shift in two of the three families. In the Judeo-Christian family, a rate change was favoured by a log Bayes factor (Log BF) of 6.59, which constitutes ‘strong evidence’ in favour of a shift. In the Islamic family, the Log BF in favour of a rate change was 2.97, constituting ‘positive evidence’. There was no evidence in favour of a shift in the Indo-Iranian family: the Log BF in favour of a shift was 0.29, considered ‘not worth more than a bare mention’ (see Table 1; Kass & Raftery, 1995).

**Table 1.** Marginal likelihood estimates (MLEs) from nested sampling for two hypotheses: H0 of no birth rate changes, and H1 of at least one birth rate change. Associated log Bayes factors (Log BFs) show strong evidence for H1 in the Judeo-Christian family and positive evidence in the Islamic family, but no substantial evidence in the Indo-Iranian family. SD = Standard Deviation of the MLE

Family	H0	SD	H1	SD	Log BF
Judeo-Christian	161.45	0.64	168.04	0.63	6.59
Islamic	6.56	0.15	9.53	0.15	2.97
Indo-Iranian	7.46	0.15	7.75	0.15	0.29

**Birth rate frequencies.** We plotted the frequencies of birth rates in the first 500 of 3,600 trees in the posterior of each sample of phylogenies (see Fig. 1) in order to identify any patterns that might correspond to birth rates before and after a shift. The distributions differed between families. In the Judeo-Christian family, the distribution was clearly bimodal, with one peak between 3 and 4 splits per 1,000 years, and another around 10. A somewhat similar pattern was visible in the Islamic family, with one clear peak around 4, together with a much lower, broader bulge in the distribution between 15 and 35. The distribution of birth rates in the Indo-Iranian family was clearly unimodal, with a single peak between 2 and 3. These distributions are consistent with support for at least one birth rate shift in the Islamic family and in the Judeo-Christian family, and a lack of support for any shifts in the Indo-Iranian family.

**Locations of birth rate shifts.** We plotted the mean posterior birth rates across all 3600 trees in the posterior on a MCC tree. These rates are summarised in Fig. 2, and are presented in detail in our Zenodo repository, https://doi.org/10.5281/zenodo.17092663, ‘Maximum clade credibility trees’. They can be interpreted as the number of binary splits expected to occur along a given lineage every 1,000 years. Points A, B, and C represent likely locations (i.e. points or intervals along the branches) of birth rate shifts.

In the Judeo-Christian MCC tree we found marked increases in the birth rate at two widely separated locations. The first of these shifts (A) clearly corresponded to the Protestant Reformation. Between 787 and 1054 Chalcedonian Christians became permanently divided into a western group that came to be known as ‘Catholic’ or ‘Roman Catholic’ and an eastern group that came to be known as ‘Eastern Orthodox’. At the time of this split the birth rate was between 3 and 4 (splits per 1,000 years). It remained within this range in the Eastern Orthodox lineage and all other Christian lineages, but there was a substantial increase in the Catholic lineage following the emergence of the Waldensians (1179–1184). By the period 1376–1382, when the Lollards emerged in England, the birth rate exceeded 5, and by the time of the Lutheran Reformation (1517–1521) it approached 10. Birth rates remained around this level in the affected clade up until 1918, with small increases in some lineages and small decreases in others. The clade within which the shift began (descendants of the Catholic Church just prior to the rise of the Lollards) consisted of 78 denominations, 74 of which still existed in 1918. The clade affected by the full magnitude of the shift (descendants of the Catholic Church just prior to the Lutheran Reformation) consisted of 75 denominations, 73 of which were extant in 1918. A large majority of these were Protestant as defined by Melton (2005). Also included were two Catholic denominations (Catholic and Old Catholic), several denominations that emerged from Protestant settings but are no longer considered Protestant (e.g. Latter-Day Saints, Unitarians), and a number of small indigenous churches that are difficult to classify as Protestant or otherwise (e.g. Indian Shaker, Ringātu).

The second increase within the Judeo-Christian MCC tree (B) was of a similar magnitude to the first, but unfolded over a longer time period. In the tenth and eleventh centuries, Rabbinic Judaism evolved from a relatively homogeneous tradition centred on the geonic academies of Babylonia to a group of independent cultural and liturgical traditions (Efron et al., 2013). A substantial increase from the Judeo-Christian baseline of 3–4 occurred in all of the new lineages that emerged at this time, but the largest uptick was in the Ashkenazi tradition of central and eastern Europe. By the time the Hasidic and Mitnagged movements emerged (1740–1772), the birth rate in this lineage approached 10, and it remained around this level until 1918. The clade within which this shift began (which included all the Rabbinic Jewish denominations in the tree that still existed in 1918) consisted of 35 denominations, 31 of which still existed in 1918. The clade most fully affected by this shift (the Ashkenazi tradition) consisted of 25 denominations, all of which still existed in 1918. Of these, 20 were Hasidic ‘courts’ or ‘dynasties’ (Biale et al., 2017). Also included in this clade are the Mitnagged or Litvak tradition (which arose in opposition to the Hasidic movement but later became aligned with it against more modernist forms of Judaism), two variants of Orthodox Judaism (Orthodox and Neo-Orthodox), and two variants of Reform Judaism (American and European).

There was only one location within the Islamic MCC tree (C) where a large increase in birth rates could be observed, and it affected a relatively small number of denominations, as can also be seen in Fig. 1 in the flat tail of birth rates in the Islamic posterior trees. However, the increase in the Islamic tree was by far the most dramatic in any of the three MCC trees. Early in their history, Sunnī Muslims became divided into a number of *madhhabs*, imperfectly translated as ‘legal schools’. The *ahl-al-ra’y*, who later became known as the Ḥanafī school, emerged in the second century AH, that is the period 722–822 CE (Kamali, 2005). In this lineage, the birth rate increased by an order of magnitude from its baseline of around 4, exceeding 20 by the period 1800–1840, and 30 by the period 1757–1905 (see our Zenodo repository, https://doi.org/10.5281/zenodo.17092663, ‘Maximum clade credibility trees: Rates schematics’). The clade affected at least partly by this shift consisted of 10 denominations, of which nine belonged to the clade that underwent the full extent of this shift. Most of these denominations can broadly be characterised as movements of reform (*islah*) or renewal (*tajdid*). Also included were the traditional Ḥanafīs (i.e. those who did not join the new movements), and two denominations (Lahore and Qadiani) belonging to the Ahmadiyya movement, whose messianic doctrines set them apart from the others. All but two of the new denominations (the Jadids of the Russian Empire and the Salafis of Egypt) originated under British rule in Northern India (Martin, 2004).

Changes in birth rates within the Indo-Iranian MCC tree were negligible, consistent with the lack of evidence for any shifts within this family.

**Sensitivity analysis.** In all of our analyses, we assumed that our sample included 80% of existing denominations belonging to the relevant traditions. This assumption seemed plausible given that our sources were encyclopaedias, which by definition aim to be comprehensive. The proportion of extinct denominations included seemed likely to vary more across phylogenies, given the patchiness of the historical record. In our main analyses, we assumed that 50% of extinct denominations were included. Assuming coverage of 99%, 25%, and 10% of extinct denominations did not substantially alter the results (see our Zenodo repository, https://doi.org/10.5281/zenodo.17092663, ‘Sensitivity analysis’).

## Discussion

Our results formally demonstrate three birth rate shifts in the history of the world religions. All three of these shifts could plausibly be linked to events in the histories of the relevant traditions, but there were some locations in the phylogenies where shifts might have been expected but were not found. We briefly consider some of the more plausible explanations for the patterns that we find, which future studies could test.

The Protestant Reformation was the most obvious candidate for being the religious equivalent of an evolutionary radiation, and so the close correspondence of the shift we detected in the Western Christian tradition to the Protestant Reformation was reassuring. The Reformation ‘proper’ is considered to have begun in 1517 when Martin Luther began to publicise his Ninety-Five Theses, but was foreshadowed by the Waldensian, Lollard, and Hussite movements of earlier centuries (Melton, 2005). Comparing the fit of the other two shifts with religious history is less straightforward because they unfolded over longer periods, but both occurred in places where they could plausibly have been expected based on the historical record. The Jewish shift began in a lineage that arose in the tenth or eleventh centuries with ‘the rise of various territorially defined Jewish subcultures’ (Efron et al., 2013, p. 151), and culminated with the emergence of the Hasidic movement in the eighteenth century. Jewish analogies with the Protestant Reformation (see e.g. Gottlieb, 2021) typically focus on Reform Judaism, which emerged slightly later, but based on our results, the impact of the Hasidic movement on Judaism’s diversity was much greater. The Islamic shift began in a lineage (corresponding to the Ḥanafī *madhhab*) that emerged in the eighth or ninth century CE, but did not begun to diversify until a 1,000 years later, when a wave of reform and revival movements swept much of the Islamic world. Many of these movements were influenced by Wahhabism, which had arisen in the previous century in what would later become Saudi Arabia (Martin, 2004). However, the Wahhabis do not form part of the shift we identified since they emerged from a different *madhhab* (Ḥanbalī). Although the Islamic shift was the least strongly supported of the three and involved the smallest number of taxa, it also involved by far the largest increase in birth rates. There were some locations where shifts might have been expected, but failed to materialise. There was virtually no sign of any birth rate increase corresponding to the *bhakti* movement (Ranade, 1900), or the so-called ‘Japanese Reformation’ of the Kamakura period (Hara, 1920).

We made a number of assumptions that necessitate caution when interpreting our results. First, we assumed a constant extinction rate for each phylogeny. As a result, we are unable to rule out the possibility that some or even all of the shifts we observed were ‘pseudoradiations’ (driven by a decrease in the extinction rate) rather than radiations in the strict sense (driven by an increase in the birth rate; Simões et al., 2016), although the timing and distribution of extinction events in our trees do not offer much support for this explanation. Second, we assumed that our sources covered each religious tradition with a similar degree of comprehensiveness. However, the encyclopaedias that we relied upon almost certainly sampled the diversity of some religious traditions more thoroughly, either due to cultural biases or simply to differences in the amount of information available. Any such discrepancy could have led to birth rates being overestimated in some clades and underestimated in others, which could conceivably have generated apparent shifts where none existed, or caused real shifts to go undetected. Gaps in the historical record could also have resulted in more lenient constraints on lineage splits in some traditions, which might have made diversification appear more gradual than it was in reality. This could have impacted our ability to detect shifts, and perhaps generated spurious shifts in cases where an apparent increase in the birth rate was really a result of improvements in records. We note, however, that there was no obvious tendency for the trees and clades where shifts were detected to be more tightly constrained (see our Zenodo repository, https://doi.org/10.5281/zenodo.17092663, ‘Raw data’). Third, we assumed that the probability of an extinct denomination being included in our sample remained constant over time, whereas it seems more likely that recently extinct denominations were overrepresented relative to long-extinct ones. This phenomenon, known as the ‘pull of the recent’ (Raup, 1979), could have generated apparent increases in the birth rate over time. Our sensitivity analyses showed our results to be robust to different assumptions about the proportion of extinct denominations included in each phylogeny, but as these analyses did not allow this proportion to vary over time, they provide only partial reassurance.

Assuming that the shifts that we uncovered were true radiations rather than pseudoradiations and were not artifacts of biases in the sources, numerous factors could explain their timing. Here we consider a small number of possibilities, which future studies could investigate. These factors fall into two broad categories, the first of which could be called ‘secular’. Perhaps the most obvious secular factor that might have promoted shifts is population growth. Each of the three shifts began within lineages that represented the most populous denomination within their respective religious traditions at the time. Catholics outnumbered all other Christians by the thirteenth century, most Muslims were Ḥanafī by the turn of the twentieth century (Barrett et al., 2001), and Ashkenazi Jews came to constitute the majority of Jews during the eighteenth century (Efron et al., 2013). The relationship between population size and rate shifts could reflect a general tendency for denominations to split when they (or their leadership cadres) reach a certain size (Fox, 2004), a need for new denominations to have a certain ‘critical mass’ of founding members, or simply a higher probability of any low-frequency event occurring in a larger population. Another factor that seems likely to have played a role is technology. There is strong historical evidence that the printing press aided the spread of the Protestant Reformation, presumably by allowing the rapid dissemination of dissenting ideas (Rubin, 2014). The timing of the Islamic and Jewish shifts is also consistent with printing technology being a factor (Efron et al., 2013; Martin, 2004). Yet another possible contributing secular factor (to the extent that it can be separated from religion) is politics. Shifts seem to be associated with times and place where centralised political authority is relatively weak or is undergoing change. The Protestant Reformation began in the Holy Roman Empire, where ‘[p]olitical decentralization frustrated censorship and control’ (Wilson, 2016, p. 266), the Hasidic movement emerged in ‘a time of increasing crisis in Polish Jewry’ associated with the abolition of Jewish self-government and the partitions of the Polish–Lithuanian Commonwealth (Biale et al., 2017), and the Islamic reform movements of the nineteenth century have been interpreted as a response to European colonialism (Martin, 2004).

The second set of possible factors that might explain religious diversification are specific religious doctrines or practices. There are some obvious candidates in the Christian and Jewish cases. McGrath and Marks (2004, pp. 1, 5) argue that the doctrine of *sola scriptura*, described as ‘an emphasis upon the supreme authority of the Bible in matters of Christian doctrine’, was responsible for Protestantism’s ‘intrinsic resistance to any concept of centralized authority corresponding to the Roman Catholic magisterium’, and that it was this resistance to religious authority that led in turn to a ‘remarkable degree of diversification’. Similarly, the Hasidic movement involved ‘a new social structure, the court of the rebbe and his followers’ (Biale et al., 2017, p. 1), which seems to have lent itself to a proliferation of independent groups led by rival rebbes. The Islamic shift is harder to explain in terms of religious factors, but one can speculate. Historically, the Ḥanafīs were perceived to have a ‘greater reliance on logic and reasoning’ than the other Sunnī schools (Martin, 2004, p. 417), a tendency which may have made them more open to reformist ideas 1,000 years later. On a broader scale, the fact that the shifts we observed were found only in Abrahamic traditions could reflect exclusivism – the claim that one’s religion is the ‘one true religion’. The Abrahamic religions have historically tended to be more exclusivist than Buddhism and Hinduism (Cobb, 1990), which may have made them less willing to compromise on matters of doctrine or religious practice, and hence more prone to fission.

There is one factor that phylogenetic methods seem unlikely to be able to model, which relates to a profound difference between biological evolution and the cultural evolution of religion. Members of religious denominations may be aware of the possibility of fission, and of the fact that their own actions can affect its probability. The denominations in our sample varied widely in the motivation and ability of their members to remain united. In some denominations, fission appears to have been viewed with indifference, or even positively. The emergence of the *madhhabs* in Sunnī Islam (Kamali, 2005), and of the *sampradāyas* in Vaiṣṇava Hinduism (Flood, 1996), seems to fall into this category. In other denominations, fission was frowned upon, but there were no mechanisms to prevent it. In early Buddhism, ‘[c]ausing a split in the Sangha [was] one of the six heinous offences, like parricide or shedding the blood of a Buddha’ (Gombrich, 2006, p. 82), and Protestants, like most other Christians, lamented schisms as ‘wounds’ in the ‘body of Christ’ (Fitzgerald, 2004). In each of these cases, however, the lack of any centralised religious authority made it difficult to translate these sentiments into counter-schismatic action. Other traditions had mechanisms that allowed them to suppress schisms, or at least reduce their incidence. Perhaps the most striking example is the community that eventually became known as the Catholic Church. Catholics maintained a centralised administrative structure and strong relationships with secular governments that often allowed them to suppress competing denominations and to manage differences that might otherwise have led to schism (Rouse & Neill, 1954), even if these efforts occasionally failed, most famously during the Protestant Reformation.

Our findings provide insight into the dynamics of religious diversification, revealing a general pattern of gradual accumulation punctuated by bursts of rapid change, paralleling processes of evolutionary radiation observed in the evolution of species. The evidence we present indicates that, on a background of prolonged periods of relative stability, a small number of transformative bursts have profoundly shaped global religious diversity. We argue these shifts likely reflect the interplay of secular and religious forces. By quantify these patterns in a rigorous statistical framework, we lay the foundation for future research to uncover the specific drivers of these dynamics, and, with that, to advance understanding of how historical, cultural, and theological factors have interacted to mould the rich diversity of religions in our world today.

## Data Availability

All data and materials used in this project are publicly available via Zenodo: https://doi.org/10.5281/zenodo.17092663.
